# New Fluorescence Probes for Biomolecules

**DOI:** 10.3390/molecules200713071

**Published:** 2015-07-20

**Authors:** Katarzyna Jurek, Janina Kabatc, Katarzyna Kostrzewska, Marlena Grabowska

**Affiliations:** Faculty of Chemical Technology and Engineering, University of Science and Technology, UTP, Bydgoszcz 85-326, Poland; E-Mails: nina@utp.edu.pl (J.K.); katarzyna.kostrzewska@utp.edu.pl (K.K.); marlena.grabowska@o2.pl (M.G.)

**Keywords:** bovine serum albumin, squarine dyes, fluorescence probe, biomolecule determination, fluorescence method

## Abstract

Steady state fluorescence measurements have been used for the investigation of interaction between the bovine serum albumin (BSA) and fluorescence probes: 3-hydroxy-2,4-bis[(3-methyl-1,3-benzoxazol-2(3*H*)-ylidene)methyl]cyclobut-2-en-1-one (SQ6), 3-hydroxy-2,4-bis[(3-methyl-1,3-benzothiazol-2(3*H*)-ylidene)methyl]cyclobut-2-en-1-one (SQ7) and 3-hydroxy-2,4-bis[(1,3,3-trimethyl-1,3-dihydro-2*H*-indol-2-ylidene)methyl]cyclobut-2-en-1-one (SQ8). The binding constant between bovine serum albumin and squarine dyes has been determined by using both the Benesi-Hildebrand and Stern-Volmer equations. The negative value of free energy change indicates the existence of a spontaneous complexation process of BSA with squarine dyes.

## 1. Introduction

The development of efficient and photostable fluorophores that absorb and emit in the visible and near-infrared spectral regions is of interest in many fields, such as optical engineering, analytical chemistry, biology, and medicine as new revolutionary tools for noninvasively and simple *in vivo* optical imaging. The visible and NIR fluorescent dyes have been often used, having the lowest autoabsorption and autofluorescence of biomolecules in the visible and NIR regions and the possibility to use low-cost excitation light sources [1,2,3,4,5,6,7,8,9,10,11,12,13].

There are several examples of BODIPY-based chemosensors. Boratriazaindacene derivatives act as chemosensors for metal cations and shows spectacular metal ion selectivity [14].

Among the metal ions showing minor fluorescence changes, whereas on addition of Hg (II) at the same concentration, the emission ratio changes more than 90-fold. Squarine dyes are ideally suited for this purpose because of their favourable optical properties associated with the peculiar zwitterionic structure, which gets perturbed with metal ions, pH, and other additives [15]. The naphthalimide and rhodamine derivatives are also reported as chemosensors for detection metal cations such as Pb(II), Cd(II), Cu(II), Ag(I) and other [16,17,18,19,20,21].

An environmental sensitive NIR fluorescent squarine dye with a thiol-reactive linker has been applied for biosensing of glucose [22].

The squarine dye molecular structures, strategies to shift the absorption and emission bands of such dyes into the visible and NIR region of the spectrum—which are outside of the self-absorption and self-emission region of biological molecules—and their spectroscopic properties are comprehensively presented eluding to their applications.

**Scheme 1:**
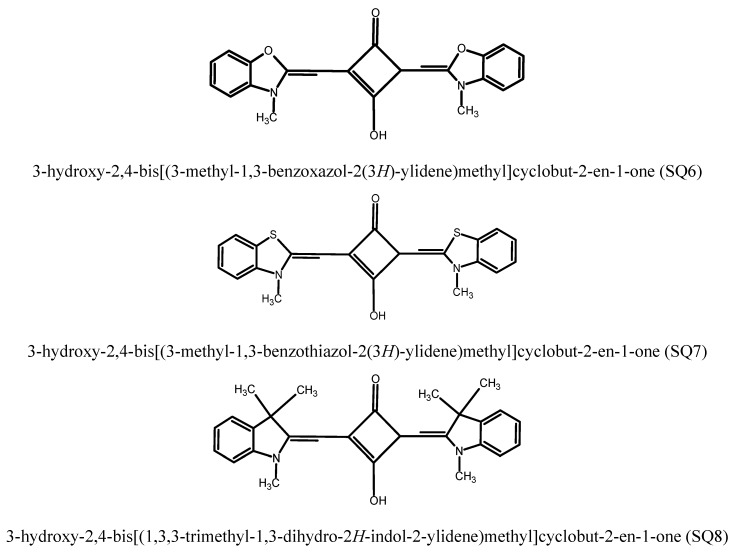
Structures of squarine dyes.

In the present paper, the series of squarine dyes are presented for their applicability as probes for detection of proteins, particularly albumins. For these purposes, the fluorescent properties of dyes in the presence of bovine serum albumin were investigated. The structure of squarine dyes selected for this study are shown in Scheme 1.

## 2. Results and Discussion

### 2.1. The Study of Squarine with BSA Association Process

The spectral data of squarine dyes in different solvents are listed in Table 1. For all dyes the red shift of the absorption band was observed with increasing of solvent polarity except water for SQ6 and dichloromethane for SQ8.

**Table 1:** Spectral data of dyes in different solvents.

Dye	Solvent	Absorption (nm)			ε_1_	ε_2_	ε_3_
		λ_1_	λ_2_	λ_3_	(×10^5^ mol^−1^·cm^−1^·L)	(×10^5^ mol^−1^·cm^−1^·L)	(×10^5^ mol^−1^·cm^−1^·L)
SQ6	Dichloromethane	283	316		0.61	0.46	
	Methanol	278	330		0.507	0.48	
	DMSO	284	343		0.39	0.437	
	Water	275	316		0.777	0.595	
SQ7	Tetrahydrofurane	625			1.25		
	Methanol	629			1.135		
	Nitromethane	631			1.25		
	Acetonitrile	635			1.19		
	DMSO	639			2.45		
SQ8	Dichloromethane	561	614	795	0.02	0.023	0.014
	Acetone	554	603	788	0.024	0.03	0.014
	Methanol	554	609	780	0.045	0.049	0.025
	Nitromethane	555	607	787	0.046	0.049	0.029
	Acetonitrile	552	602	785	0.027	0.029	0.013
	DMSO	568	611		0.049	0.055	

**Figure 1:**
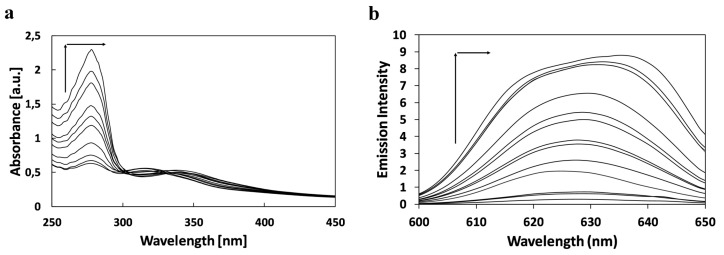
Effect of increasing concentration of BSA on the absorption (**a**) ([BSA] = 0, 2, 3, 7, 13, 16, 20, 27, 30, 40 µM) and fluorescence emission (**b**) ([BSA] = 0, 2, 3, 7, 13, 16, 20, 27, 30, 40, 80, 100, 140 µM) spectra of SQ6 ([SQ6] = 150 µM).

As shown in Figure 1, the changes in absorption and emission spectra of SQ6 as an effect of increasing concentration of bovine serum albumin results in an increase of the absorbance at 277 without changing the position of the first absorption maximum and with a gradual red shift of the second absorption maximum from 316 nm to 350 nm. The similar effect was observed on absorption spectra of SQ7 and SQ8. The absorbance increasing at 277 nm without a shift of maxima and with a red shift from 620 to 640 nm was observed for the SQ7. The fluorescence spectra showed a gradual shift of emission maxima from 622 to 637 nm (SQ6), 675 to 732 nm (SQ7) and 680 to 650 nm (SQ8), respectively.

The binding constant of monomeric dyes with albumin, *K* in M^−1^ and the binding parameters were determined basing on the equilibrium [Equation (1)]:

(1) $$\begin{array}{c} SQ+BSA\overset{k}{↔}SQ:BSA\\ K=\frac{{\left[SQ:BSA\right]}_{b}}{{\left[SQ\right]}_{f}{\left[BSA\right]}_{f}} \end{array}$$

where [SQK:BSA], [SQK] were concentration of bounded and free dye, respectively. The concentration of each components can be expressed in terms of fluorescence intensity. Assuming that the concentration of protein-dye complex is very low compared to the free BSA concentration, the modification of Benesi-Hildenbrand equation can be written as follows (Equation (2)) [23,24].

(2) $$\frac{1}{I–I_{0}}=\frac{1}{I_{1}–I_{0}}+\frac{1}{\left(I_{1}–I_{0})K[BSA\right]}$$

where *I*_0_ and *I* is intensity of dye in absence and presence of bovine serum albumin.

**Figure 2:**
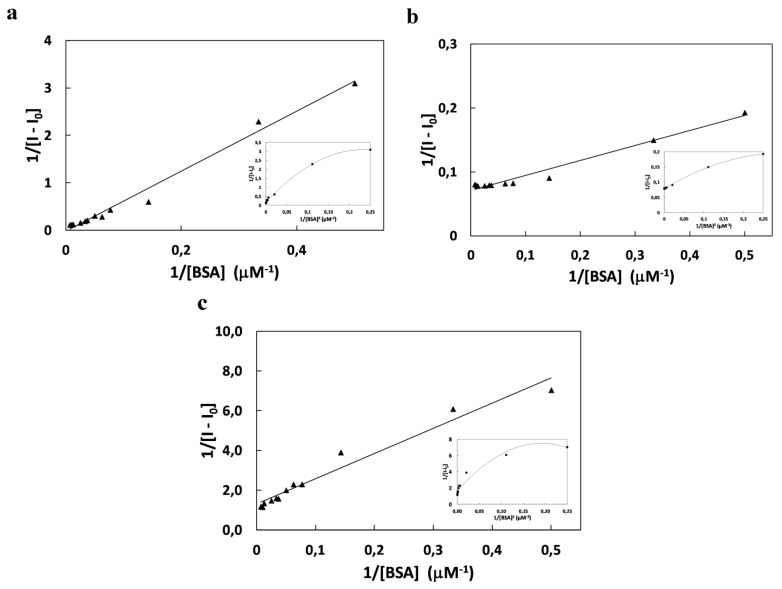
Benesi *−* Hildebrandt plot of 1/*I − I*_0_ *vs.* 1/[BSA] for binding of SQ6 (**a**); SQ7 (**b**); SQ8 (**c**) with inserted correlation 1/*I − I*_0_ with 1/[BSA]^2^ for binding process of squarine dyes with BSA.

The plots of 1/(I − I_0_) *vs.* 1/[BSA] presented on Figure 2b,c shows the linear fitting is less correlated than parabolic fitting which suggests to 1:2 complexation between fluorophore and protein with very high binding constants 2.58 ´ 10^4^ (SQ6), 3.03 #x00B4;; 10^6^ (SQ7), 1.02 #x00B4;; 10^6^ M^−2^ (SQ8), respectively. From the value of binding constants, it is known that the all squarine dyes bind very strongly with BSA. The calculated negative free energy change, Δ*G* (Table 2) from the *K* value for squarine:BSA binding process indicated that spontaneous complexation occurs.

### 2.2. Study of Quenching Process of BSA by Squarine Dyes

The intrinsic quenching effect of bovine serum albumin by squarine dyes was studied. The decrease of fluorescence intensity of BSA with increasing concentration of squarine dye was observed. The effective quenching constant for the accessible fluorophore, *K_a_* and fraction of accessible fluorescence, *f_a_* were found using the Lehrer equation (Equation (3)) [25] presented below:

(3) $$\frac{I_{0}}{I_{1}–I}=\frac{1}{f_{a}K_{a}[SQ]}+\frac{1}{f_{a}}$$

where *I*_0_ and *I* are the intensities of BSA in the absence and presence of the quencher (squarine dye) at concentration [SQ].

The fluorescence data were analyzed also using the Stern-Volmer equation (Equation (4)):

(4) $$\frac{I_{0}}{I}=1+K_{SV}[SQ]$$

where: *K_SV_* is Stern-Volmer constant of SQ:BSA complex.

**Figure 3:**
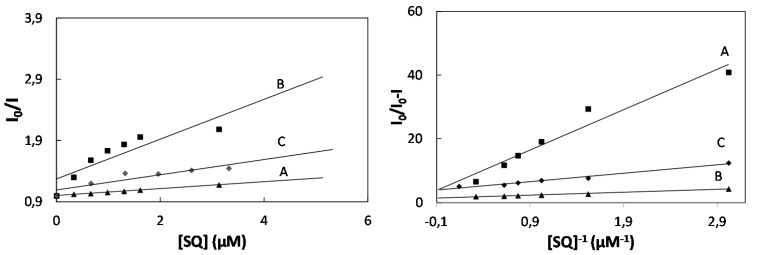
(**Left**) Stern-Volmer plot and (**Right**) Lehrer plot for the quenching of BSA by SQ6 (A), SQ7 (B) and SQ8 (C).

The Lehrer and Stern-Volmer plots are presented in Figure 3.

As shown in Figure 3, the effect of increasing concentration of squarine dyes have linear dependence which provided to determination of quenching constant for the accessible fluorophore, *K_a_* and Stern-Volmer constant of SQ:BSA complex, *K_SV_* (Table 2).

The quenching constant and number of binding sites were calculated from Stern-Volmer equation (Equation (5)) presented below:

(5) $$log\frac{I_{0}–I}{I}=logK_{b}+nlog[SQ]$$

where *I*_0_ and *I* are the fluorescence intensities of BSA in absence and presence of SQ, *K_b_* and *n* are quenching constant and number of binding sites, respectively. The quenching parameters of squarine dyes with bovine serum albumin are collected in Table 2.

**Table 2:** Binding parameters for interaction of squarine dyes with BSA.

	K (×10^4^ M^−2^)	K_a_ (×10^4^ M^−1^)	K_SV_ (×10^4^ M^−1^)	K_b_ (×10^4^ M^−1^)	*n*	ΔG (kJmol^−1^)
SQ6	2.58	40.4	5.65	1.49	0.90	−25.17
SQ7	303	18,7	32.5	0.18	0.57	−36.98
SQ8	102	160	12.8	0.04	0.56	−34.29

**Figure 4:**
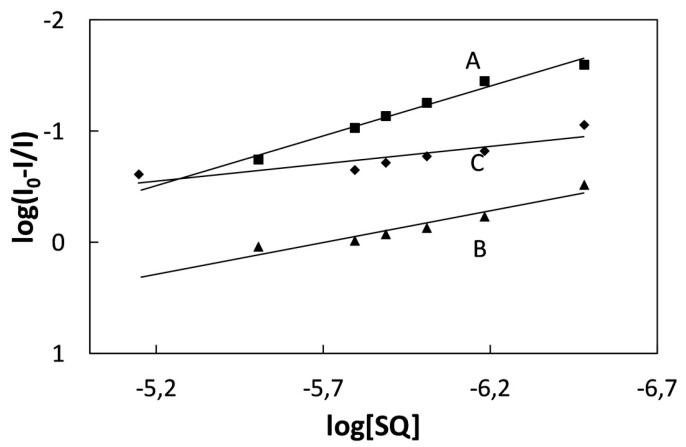
Modified Stern-Volmer plots of binding parameters BSA with squarine dyes: SQ6 (A), SQ7 (B), SQ8 (C).

As shown in Figure 4, the quenching constant increases with following series of dye: SQ8, SQ7, and SQ6. From the increasing concentration of squarine dye the number of binding sites of quenching process is lower than 1 for SQ7 and SQ8 which confirms that the 1:2 complexation process of bovine serum albumin with squarine dyes.

## 3. Experimental Section

### 3.1. Synthesis

The substrates used for synthesis of squarine dyes were purchased from Sigma-Aldrich (Poznań, Poland) and were used without further purification. The squarylium dyes: 3-hydroxy-2,4-bis[(3-methyl-1,3-benzoxazol-2(3*H*)-ylidene)methyl]cyclobut-2-en-1-one (SQ6), 3-hydroxy-2,4-bis[(3-methyl-1,3-benzothiazol-2(3*H*)-ylidene)methyl]cyclobut-2-en-1-one (SQ7) and 3-hydroxy-2,4-bis[(1,3,3-trimethyl-1,3-dihydro-2*H*-indol-2-ylidene)methyl]cyclobut-2-en-1-one (SQ8) were synthesized by the methodology described in work [4].

### 3.2. Materials

Distilled water, Tris-HCl buffer, bovine serum albumin (BSA) were purchased from Sigma-Aldrich (Poznań, Poland). Solvents were of the analytical grade.

### 3.3. Preparation of Stock Solutions of Dyes and Proteins

The 2 #x00B4;; 10^−3^ M dye stock solutions were prepared by dilution of the dye in DMSO. Stock solutions of proteins BSA 0.15 mM were prepared by dissolving in 0.05 M Tris-HCl buffer (pH 8.0).

### 3.4. Preparation of Working Solutions

The working solutions of free dyes were prepared by dilution of the dye stock solution in 0.05 M Tris-HCl buffer. Working solutions of the dyes in the presence of proteins were prepared by addition of the dye stock solution to the protein stock solution. The concentrations of dye and protein in working solutions amounted to 2 #x00B4;; 10^−6^ M and 1.4 #x00B4;; 10^−4^ M, respectively. All working solutions were prepared immediately before the experiments. The 1 #x00B4;; 10^−4^ and 5 #x00B4;; 10^−5^ M titration solutions of free dyes and BSA were prepared by dilution of the dye and protein stocks solution in 0.05 M Tris-HCl buffer.

### 3.5. Spectroscopic Measurements

The effect of an increasing concentration of BSA on absorption spectra of dyes were measured on a Cary 50 spectrophotometer (Varian, Warsaw, Poland). Working solution in presence of BSA was prepared by addition of dye stock solution to the protein solution. The concentration of dye in working solution was equal 1.5 #x00B4;; 10^−4^ M. The concentration of BSA were: 0, 2, 3, 7, 13, 16, 20, 27, 30, 40 µM, respectively.

The effect of an increasing concentration of BSA on fluorescence spectra of fluorophores was study on the same working solution in the addition of following concentration of BSA: 0, 2, 3, 7, 13, 16, 20, 27, 30, 40, 80, 100, 140 µM. Fluorescence spectra were taken on FLS 920 Spectrofluorimeter (Edinberg Instruments). Spectroscopic measurements were performed in standard quartz cell (1 × 1 cm).

The parameter of binding process of squarines with BSA were determinated by titration process of BSA solution (5 #x00B4;; 10^−5^ M) with increasing amounts of dye: 0.66, 1.32, 1.96, 2.60, 3.32, 6.25, 14.30 µM. The fluorescence spectra were recorded on FLS 920 Spectrofluorimeter (Edinberg Instruments, Livingstone, UK).

## 4. Conclusions

The series of squarine dyes was characterized in the presence of bovine serum albumin using fluorescence spectroscopy. The studied dyes presented a very high binding constant which confirms the very strong binding of fluorophore with bovine serum albumin. The negative free energy change indicated that an interaction of squarine dyes with BSA is a spontaneous complexation process. This indicates the potential of the studied squarine dyes as a spectral and fluorescence probe for BSA.
